# The complete chloroplast genome sequence of *Magnolia mexicana* DC. (Magnoliaceae) from Central America

**DOI:** 10.1080/23802359.2020.1715854

**Published:** 2020-01-24

**Authors:** Guang-Ning Liu, Bin-Bin Liu, Jun Wen, Yu-Bing Wang

**Affiliations:** a College of Architecture and Urban Planning, Tongji University, Shanghai, China;; b State Key Laboratory of Systematic and Evolutionary Botany, Institute of Botany, Chinese Academy of Sciences, Beijing, China;; c Department of Botany, National Museum of Natural History, Smithsonian Institution, Washington, USA;; d Key Laboratory of Three Gorges Regional Plant Genetics and Germplasm Enhancement (CTGU)/Biotechnology Research Center, China Three Gorges University, Yichang, China

**Keywords:** Chloroplast genome, *Magnolia mexicana*, *Magnolia*, Magnoliaceae, phylogeny

## Abstract

The chloroplast genome of the *Magnolia* species from Central America has never been reported. With its local use for food flavoring, medicine, and wood, *M. mexicana* has been of good economic importance. In the present study, the complete chloroplast genome of *M. mexicana* was assembled via the genome skimming data. As a typical quadripartite structure, the plastome of *M. mexicana* with 159,906 bp in length includes two inverted repeats (26,554 bp) separated by a small single copy region (18,761 bp) as well as a large single copy region (88,037 bp). This chloroplast genome consists of 131 different genes, including 86 protein coding genes (CDS), eight rRNA genes, and 37 tRNA genes. The maximum likelihood phylogenetic analysis showed that *M. mexicana* from Central America was closely related to an evergreen species, *M. odoratissima* from East Asia.

The *Magnolia* family, Magnoliaceae, consists of ca. 300 species disjunctly distributed from Eastern and Southeastern Asia to the New World (Xia et al. 2009), with many species used as ornamentals, medicine, and spices. However, the phylogenetic relationships of the *Magnolia* species from Central America have never been well tested. *Magnolia mexicana* DC. has been of good economic importance because of its local use for food flavoring, medicine, and wood. In this study, we assembled the complete chloroplast genome of *M. mexicana* via the genome skimming method (Zhang et al. 2016). Our aims were to characterize the organization of the plastome and clarify the phylogenetic position of *M. mexicana*.

The voucher for *Magnolia mexicana* was deposited in the United States National Herbarium (US, wen 8726), and it was collected from Chiapas, Mexico by Jun Wen. Total genomic DNA was extracted from the silica-gel dried leaves using the Qiagen DNeasy^®^ plant mini-kit (Qiagen Gmbh, Hilden, Germany), and the library was prepared with the NEBNext^®^ Ultra™ II DNA Library Prep Kit in the Laboratories of Analytical Biology (LAB), National Museum of Natural History (NMNH), Smithsonian Institution, USA. The library was sequenced in the Novogene UC Davis Sequencing Center, Davis, California, USA using an Illumina HiSeq 2500 instrument. Paired-end reads of 2 × 150 bp were generated. We removed the adaptors introduced by Illumina sequencing using cutadapt 2.4 (Martin 2011) with AGATCGGAAGAGC as the forward and the reverse adaptor. The results were checked for quality control with FastQC 0.11.8 (Andrews 2018). We assembled the chloroplast genome using NOVOPlasty 3.6 (Dierckxsens et al. 2017), which has been used successfully in several lineages of angiosperms, such as in Araliaceae (Liu and Wen 2019) and Rosaceae (Liu et al. 2019). The assembled plastid genomes were annotated using Geneious Prime (Kearse et al. 2012) with a well-annotated sequence from NCBI as a reference (NC_037005). The best-fit nucleotide substitution models for the plastid datasets were estimated by PartitionFinder2 (Lanfear et al. 2016), and the maximum likelihood (Stamatakis, 2006) tree was inferred by IQ-TREE v.1.6.9 (Nguyen et al. 2015). The annotated plastid sequence has been submitted to GenBank with the accession number MN700657.

The complete chloroplast genome has been found to have a typical quadripartite structure with 159,906 bp in length, including two short inverted repeats (IRa and IRb: 26,554 bp) separated by a small single copy region (SSC: 18,761 bp), and a large single copy region (LSC: 88,037 bp). The complete chloroplast genome of *M. mexicana* encoded 131 genes including 86 protein coding sequences (CDS), eight rRNA genes, and 37 tRNA genes. Among all these genes, a single intron was detected in 17 genes, while two genes (*clpP* and *ycf3*) were found to have two introns each.

Our phylogenetic analysis (Figure 1) showed that Central American evergreen species, *Magnolia mexicana*, was closely related to an evergreen species, *Magnolia odoratissima* Y.W.Law and R.Z.Zhou from East Asia, and this relationship suggests a strong biogeographic connection between tropical Asia and the Neotropics (Valcárcel and Wen 2019).

**Figure 1.**
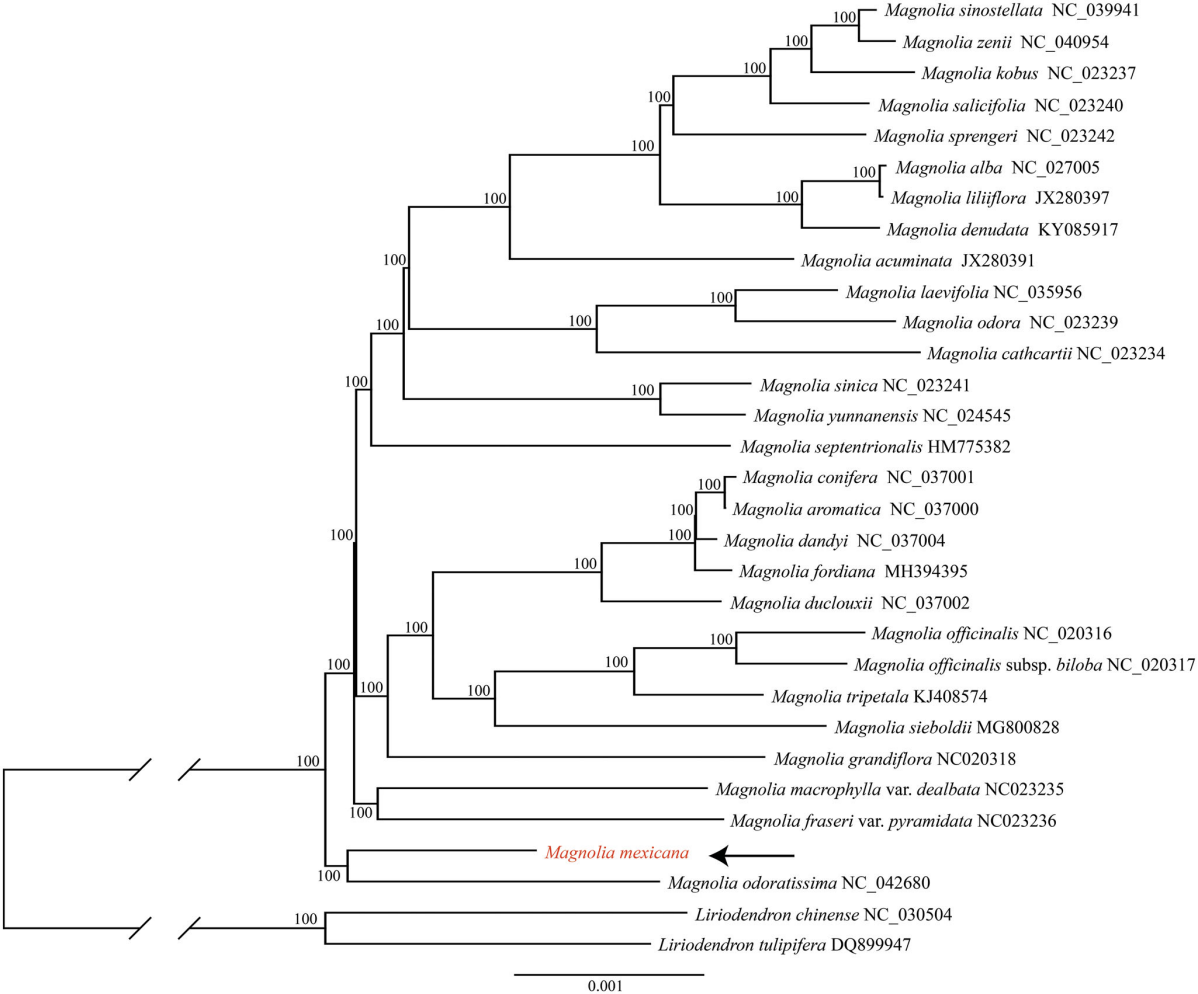
The phylogenetic placement of *Magnolia mexicana* in the framework of Magnoliaceae resolved by maximum likelihood methodbased on the complete chloroplast genome. The numbers associated with the branches are ML bootstrap value.
